# Accuracy of M2BPGi, compared with Fibro Scan®, in analysis of liver fibrosis in patients with hepatitis C

**DOI:** 10.1186/s12876-017-0618-5

**Published:** 2017-05-10

**Authors:** Hongqin Xu, Wenli Kong, Lei Liu, Xiumei Chi, Xiaomei Wang, Ruihong Wu, Xiuzhu Gao, Huan Wang, Limei Qu, Yue Qi, Yu Pan, Junqi Niu

**Affiliations:** 1 Department of Hepatology, First Hospital of Jilin University, Jilin University, Changchun, 130021 China; 2 Ministry of Education Key Laboratory of Zoonosis, Changchun, 130061 China; 3 Jilin Province Key Laboratory of Infectious Diseases, Laboratory of Molecular Virology, Changchun, 130061 China; 4 Department of Pathology, First Hospital of Jilin University, Jilin University, Changchun, 130021 China

**Keywords:** Chronic hepatitis C, Liver fibrosis, Mac-2 Binding Protein Glycosylation isomer, Cirrhosis

## Abstract

**Background:**

Mac-2 Binding Protein Glycosylation isomer (M2BPGi) is a novel serological glyco-biomarker for staging liver fibrosis. Here, we aimed to evaluate the efficiency of serum M2BPGi in identifying liver fibrosis stages in Chinese patients with chronic hepatitis C infection.

**Methods:**

Serum M2BPGi levels were evaluated in 680 patients with chronic hepatitis C and 164 healthy controls who underwent the Fibro Scan® test of liver fibrosis. The diagnostic accuracy of serum M2BPGi values was compared to that of other fibrosis markers, including Fibro Scan®, the aspartate transaminase to platelet ratio index (APRI), the fibrosis index based on four factors (FIB4), and the gamma-glutamyltranspeptidase to platelet ratio (GPR).

**Results:**

Among the chronic hepatitis C patients, the median serum M2BPGi level increased with increasing fibrosis score as follows: 0.88 (≤F2), 1.70 (F2/F3), and 5.68 (cirrhosis). M2BPGi concentrations could also distinguish between healthy controls (0.38 ± 0.24) and hepatitis C patients (1.57 ± 2.28). After adjusting for potential confounders, M2BPGi was the most significant factor associated with the liver stiffness measurement (effect size = 0.275, *P* < 0.001). The optimum cutoff values of serum M2BPGi for patients with F2 and F4 were 0.945 and 1.355, respectively. The area under the curve of serum M2BPGi for prediction of significant fibrosis (F ≥ 4) using was comparable to that of APRI (0.892 vs. 0.873), while it was superior to that of other alternative markers, including FIB4 (0.818) and GPR (0.851). Compared with other non-invasive markers, M2BPGi had the greatest specificity for diagnosing cirrhosis and cirrhosis in hepatitis C patients.

**Conclusions:**

Our results suggest that the level of serum M2BPGi would be a simple and reliable diagnostic tool for identifying liver fibrosis stage in Chinese patients with chronic hepatitis.

## Background

Hepatitis C virus (HCV) is a major cause of liver disease worldwide. HCV establishes persistent infection that often results in chronic hepatitis followed by liver fibrosis, cirrhosis, from which hepatocellular carcinoma arises. The stage of chronic liver disease is mainly evaluated based on the degree of liver fibrosis. Therefore, assessment of the degree of liver fibrosis is important in clinical practice. Liver biopsy is the gold standard method for evaluating the degree of liver fibrosis [1]. However, the invasiveness nature of liver biopsy makes it un-practical approach, especially for patients who require follow-up [2].

Recently, glycoprotein-based biomarkers (glyco-biomarker) have been emerged as novel disease biomarkers. Mac-2 Binding Protein Glycosylation isomer (M2BPGi), which is also named hyperglycosylated Wisteria floribunda agglutinin-positive Mac-2 binding protein (WFA+ -M2BP) is a new serological glyco-biomarker that has been recently developed for predicting the stage of liver fibrosis [3, 4]. This technical approach may be applicable to the development pipeline for a wide variety of glyco diagnostic tools.

Developed in France by Echosens, transient elastography (Fibro Scan®) is a novel, noninvasive technique that is used to assess the degree of liver fibrosis. The Vibration Controlled Transient Elastography™ system (VCTE™) implemented on the Fibro Scan®[5] is the most validated and commonly used elastography method worldwide and has been approved in China by the China Food and Drug Administration [6, 7]. Data suggest VCTE™ is reliable in diagnosing cirrhosis in patients with chronic liver disease [8], liver fibrosis in autoimmune hepatitis [9] advanced fibrosis in patients with alcoholic liver disease [10], significant fibrosis in patients with chronic hepatitis C [11], and in biliary diseases [12]. In recent years, the liver stiffness measurement (LSM) which is resulting from transient elastography has been applied to predict liver fibrosis in many hospitals in China [13, 14].

This study aimed to (1) identify optimal serum M2BPGi cut-off values for evaluating the stage of liver fibrosis in Chinese patients with chronic hepatitis C, (2) identify the factors independently associated with M2BPGi, and (3) compare the diagnostic value of M2BPGi versus the fibrosis biomarkers aspartate transaminase to platelet ratio index (APRI), fibrosis index based on four factors (FIB-4), and gamma-glutamyltrans peptidase to platelet ratio (GPR).

## Methods

### Patients

We retrospectively reviewed 1001 hepatitis C patients and 164 healthy controls who underwent Fibro Scan® testing and color Doppler ultrasound at The First Hospital of Jilin University (Changchun, China). Of these, 321 patients who had fatty liver, which might influence the value of the Fibro Scan®, were excluded from the study. The study was approved by the Ethics Committee at the First Hospital of Jilin University, Changchun, China and the written informed consent was obtained from each patient enrolled in this study.

### LSM

Fibro Scan® (Echosens, France) was performed by skillful operator to assess LSM value. Ten LSM values were recoreded and the median value calculated by the statistics analyze system was called as the final score. According to Tsochatzis [15] et al, the liver stiffness cut-offs were staged on a scale of 0–4 according to Fibro Scan® given as 7.6 (range 5.1–10.1), 10.9 (8.0–15.4), and 15.3 (11.9–26.5) kPa for stages F2, F3, and F4 in hepatitis C patients respectively.

### Direct measurement of serum M2BPGi

Serum M2BPGi levels were quantified by a lectin-antibody sandwich immunoassay using HISCL-5000 immunoanalyzer (Sysmex Co., Hyogo, Japan) [4, 16]. M2BPGi values conjugated to WFA were indexed with scored values using the following equation: Cutoff index = ([M2BPGi] sample − [M2BPGi] NC)/([M2BPGi] PC −[M2BPGi] NC), where [M2BPGi] sample is the M2BPGi level in the serum sample, PC is the positive control, and NC is negative control. The positive control was supplied as a calibration solution preliminarily standardized to yield a cutoff value of 1.0.

### Surrogate serum markers

On the same day that Fibro Scan® examination was performed, blood specimens were obtained and subjected to hematological and biochemical examinations. Hyaluronic acid level, type 4 collagen (CIV) level, laminin (LN) level, platelet count, aspartate aminotransferase (AST) level and alanine aminotransferase (ALT) level were determined in the same laboratory. APRI, FIB-4, and GPR scores were calculated based on the following formulae:

$$ \mathrm{APRI} = \left(\left[\mathrm{AST}/\mathrm{ULN}*\right]/\mathrm{platelet}\ \mathrm{count}\right) \times 100; $$

$$ \mathrm{FIB}-4 = \left(\mathrm{age} \times \mathrm{AST}\right)/\left(\mathrm{platelet}\ \mathrm{count} \times \surd \mathrm{ALT}\right); $$

GPR = GGT/platelet count, where GGT is γ-glutamyltransferase.

### Statistical analysis

Differences between continuous variables of paired samples were analyzed using Mann-Whitney U test. Receiver operating characteristic (ROC) curve and area under the curve (AUCs) were used to determine the optimal liver stiffness cutoffs for fibrosis staging. The optimal liver stiffness cutoffs were determined based on the optimal sensitivity, specificity, positive predictive value (PPV), and negative predictive value (NPV). Statistical analysis of the differences between the AUCs was based on the theory of generalized U-statistics. The *P* <0.05 was considered significant.

## Results

### Baseline clinical characteristics of patients

The baseline clinical characteristics of enrolled patients are shown in Table 1. The median age was 54.9 years with a male predominance (68.4%) in the hepatitis C group, while the median age was 56.5 years with a female predominance (54.7%) in the control group. Two hundred ninety-one patients were treated with interferon (IFN) + ribavirin (RBV). The median (interquartile range, IQR) values for the body mass index (BMI), APRI, FIB-4 index, AST/ALT ratio, and GPR were 23.4 (21.5–25.5), 0.48 (0.32–1.01), 2.81 (1.87–4.65), 1.44 (1.07–2.69), and 0.18 (0.09–0.47), respectively, in the hepatitis C-infected group. The fibrosis stage was < F2 (LSM < 7.6) for 437 cases (64.3%), F2 or F3 (7.6 ≤ LSM < 15.3) for 175 cases (25.7%), and F4 (LSM > 15.3) for 68 cases (10.0%). BMI, APRI, FIB-4 index, AST/ALT ratio, GPR, and M2BPGi level differed significantly between hepatitis C patients and healthy controls.

**Table 1:** Patients’ clinical characteristics and laboratory data

Features	Cases (*n* = 680)	Healthy (*n* = 164)	*P* value
Male/Female, n	465/215	76/88	
Age (years)	54.9 ± 11.0	56.8 ± 14.8	0.02
IFN therapy N (%)	291 (42.8%)	0 (0.00%)	
Habitual alcohol intake N (%)	275 (40.4%)		
Body mass index	23.6 ± 3.1	24.0 ± 3.1	0.114
Platelet count (×10^9^/l)	166.7 ± 74.8	228.3 ± 45.1	<0.001
AST (15–46 IU/l)	49.2 ± 39.9	26.4 ± 10.2	<0.001
ALT (0–40 IU/l)	37.7 ± 43.8	23.7 ± 15.1	0.011
GGT (12–73 IU/l)	63.7 ± 105.3	27.2 ± 27.7	<0.001
AFP (0–6.2 IU/l)	7.46 ± 33.9		
HCV genotype N (%)			
1b	126 (18.5%)		
2a	94 (13.8%)		
1b/2a	2 (0.3%)		
Unknown	459 (67.4%)		
Fibrosis markers			
APRI	1.06 ± 2.72	0.30 ± 0.13	<0.001
FIB4	4.84 ± 12.05	1.33 ± 0.55	<0.001
AST/ALT ratio	2.62 ± 2.80	1.45 ± 1.43	<0.001
GPR	0.58 ± 1.21	0.12 ± 0.12	<0.001
M2BPGi	1.57 ± 2.28	0.38 ± 0.23	<0.001
Fibrosis stage (0 ~ 1/2 ~ 3/4)	437/175/68	155/9/0	

### Correlation between M2BPGi and LSM in patients with HCV infection

**Fig. 1:**
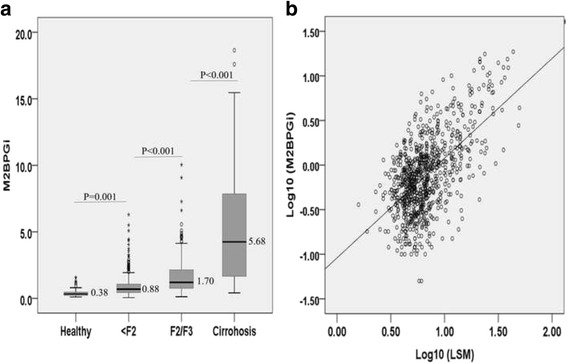
**a** The serum Wisteria floribunda agglutinin-positive Mac-2 binding protein (M2BPGi) values for each fibrosis stage. The *top* and *bottom* of each *box* represent the first and third quartiles, respectively, with the height of the box representing the interquartile range, covering 50% of the values. The line across each box represents the median. The whiskers show the highest and lowest values. **b** Correlation of log10 (M2BPGi) values with log10 (LSM)

Among chronic hepatitis C patients, the median level of serum M2BPGi was positively associated with fibrosis stage as 0.88 (<F2), 1.70 (F2/F3), and 5.68 (F4). As the median serum M2BPGi level was 0.38 in healthy controls, there was also a significant difference between healthy controls and hepatitis C patients (*P* < 0.001). Figure 1a shows a box-plot of liver fibrosis stage (LSM) vs. M2BPGi level. Moreover, significant differences were noted for M2BPGi level among the fibrosis stages. The differences were significant (*P* < 0.001) between healthy controls and chronic hepatitis C patients and between F2/3 and F4 stages (*P* < 0.001). Using Spearman rank correlation analysis, a positive correlation between LSM and M2BPGi level (rho = 0.504, *P* < 0.001) was observed (Fig. 1b).

### Variables associated with LSM obtained by Fibro Scan®

Correlation between LSM and baseline demographic (age and gender), hematological and biochemical characteristics (platelet count, total protein, albumin, ALT, AST, alkaline phosphatase, γ-glutamyltransferase, bilirubin, FIB-4 index, APRI, AST/ALT ratio, and GPR) was also examined (Table 2). LSM was significantly correlated with ALT (rho = 403, *P* < 0.001), AST (rho = 0.499, *P* < 0.001), alkaline phosphatase (ALP; rho = 0.309, *P* < 0.001), GGT (rho = 0.432, *P* < 0.001), total bilirubin (rho = 0.274, *P* < 0.001), FIB-4 (rho = 0.412, *P* < 0.001), APRI (rho = 0.536, *P* < 0.001), and all histological results and was inversely correlated with platelet count (rho = -0.386, *P* < 0.001) and AST/ALT ratio (rho = -0.141, *P* < 0.001). Gender, age, cigarette smoking, platelet count, total protein, albumin, liver function markers (AST, ALT, ALP, GGT, TB, HA, LN, α-fetoprotein) and M2BPGi level were associated with LSM in univariate analysis. Subsequently these factors were entered in logistic regression multivariable analysis. This analysis identified platelet count (effect size = -0.07, P ≤ 0.05),total protein level (effect size = -0.08, P ≤ 0.05), ALP (effect size = 0.116, P ≤ 0.05), GGT (effect size = 0.13, *P* < 0.001), total bilirubin (effect size = 0.111, *P* = 0.001), HA (effect size = 0.137, *P* < 0.001), CIV (effect size = 0.099, P ≤ 0.05), AFP (effect size = 0.069, P ≤ 0.05), and M2BPGi (effect size = 0.275, *P* < 0.001) levels as independent factors that were significantly associated with LSM (Table 2). Notably, serum level of M2BPGi was the most significant factor associated with LSM.

**Table 2:** Variables associated with the LSM according to liner regression analyses

Characteristic	Correlation analysis		liner regression analysis	
	rho	*P* value	Effect size	*P* value
Age	0.188	<0.001	0.015	0.606
Gender	−0.099	0.01	−0.026	0.404
BMI	0.038	0.318	-	-
Laboratory findings				
Platelet count	−0.386	<0.001	−0.07	0.02
Total protein	0.194	<0.001	−0.08	0.032
Albumin	−0.18	<0.001	−0.071	0.073
ALT	0.403	<0.001	0.017	0.732
AST	0.499	<0.001	0.008	0.893
ALP	0.309	<0.001	0.116	0.001
GGT	0.432	<0.001	0.13	<0.001
Total Billirubin	0.274	<0.001	0.111	0.001
HA	0.338	<0.001	0.137	<0.001
LN	0.234	<0.001	0.03	0.38
CIV	0.34	<0.001	0.099	0.003
AFP	0.379	<0.001	0.069	0.033
Fibrosis markers				
FIB-4 indext	0.412	<0.001	-	-
APRI	0.536	<0.001	-	-
AST/ALT ratio	−0.141	<0.001	-	-
GPR	0.487	<0.001	-	-
M2BPGi	0.504	<0.001	0.275	<0.001

### Comparison of AUCs and cut-off values for fibrosis markers

ROC analyses were carried out to evaluate the diagnostic accuracy of serum M2BPGi, FIB-4 index, APRI, and GPR for fibrosis stage in chronic hepatitis C. The calculated AUC, optimal cut-off value, sensitivity, specificity, PPV, and NPV for each fibrosis stage are listed in Table 3. The AUCs were 0.774 and 0.892 for ≥ F2 and F4, respectively. The optimal cut-off values predicted fibrosis stages ≥ F2 and F4 were 0.945 and 1.355, respectively.

**Table 3:** Diagnostic performance of M2BPGi in 680 patients with chronic hepatitis C

Fibrosis stage		Cutoff	AUC	Sensitivity	Specificity	*P* value
≥F2	M2BPGi	0.945	0.774 (0.736, 0.812)	0.745	0.694	Reference
	APRI	0.635	0.787 (0.750, 0.824)	0.708	0.799	>0.05
	FIB4	3.145	0.702 (0.661, 0.743)	0.650	0.686	<0.001
	GPR	0.221	0.764 (0.726, 0.801)	0.704	0.716	>0.05
≥F4	M2BPGi	1.355	0.892 (0.851, 0.933)	0.868	0.786	Reference
(Cirrhosis)	APRI	0.885	0.873 (0.835, 0.910)	0.838	0.783	>0.05
	FIB4	4.395	0.818 (0.768, 0.868)	0.750	0.775	<0.05
	GPR	0.291	0.851 (0.808, 0.894)	0.882	0.691	>0.05

**Fig. 2:**
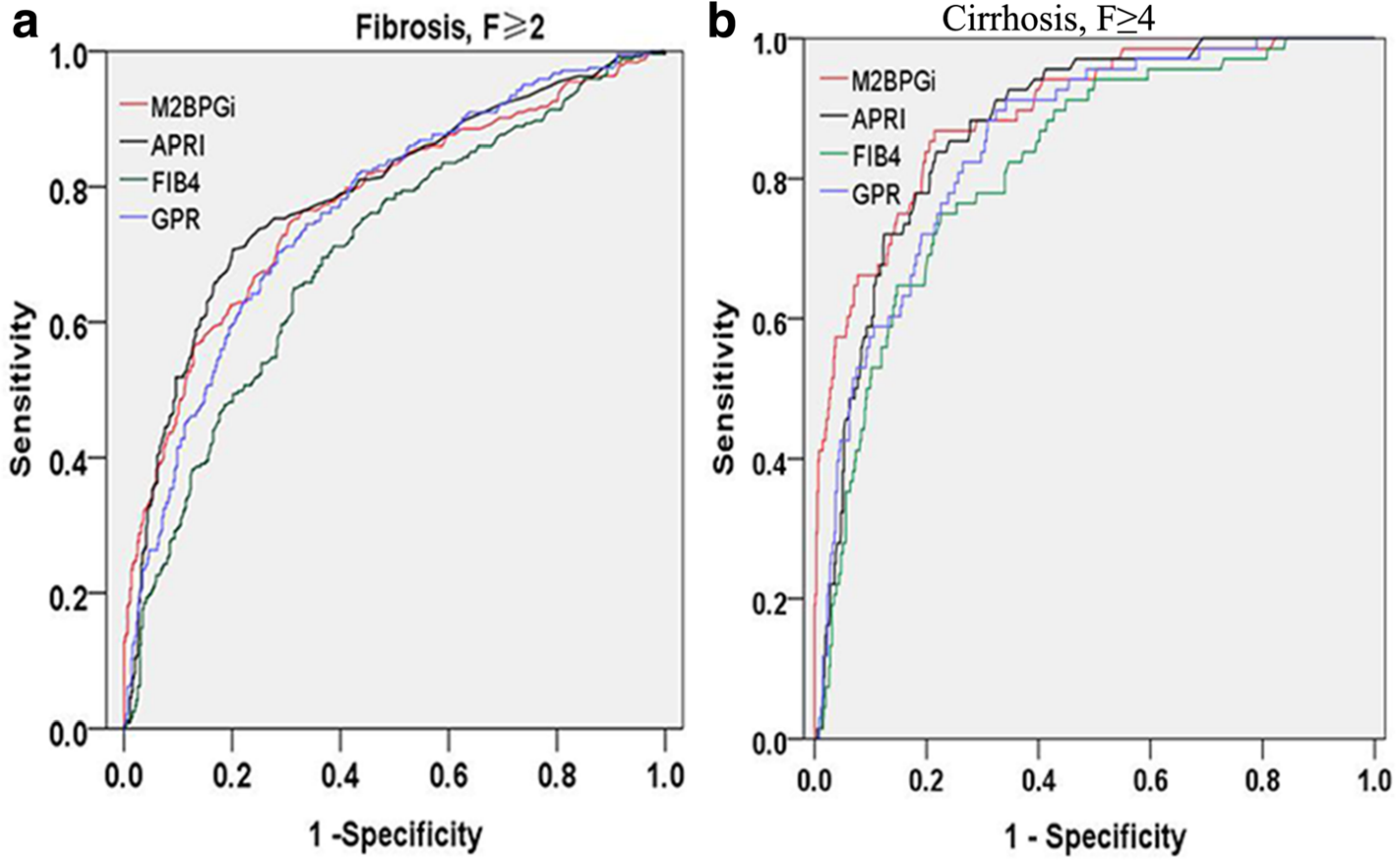
The diagnostic capabilities of the M2BPGi values for assessing the stage of liver fibrosis. The AUCs for serum M2BPGi in diagnosing liver fibrosis were as follows: **a** 0.774 for stage ≥ F2; **b** 0.892 for stage ≥ F4

The ROC curves for M2BPGi, FIB-4 index, APRI, and GPR for predicting severe fibrosis (≥F2) and cirrhosis (F4) are shown in Fig. 2a–b. The AUC of M2BPGi for predicting significant fibrosis (≥F2) was significantly greater than those for the FIB-4 index (AUC = 0.702, *P* < 0.001), and similar to those for GPR (AUC = 0.764, *P* > 0.05) and APRI (AUC = 0.787, *P* > 0.05). For estimating cirrhosis, the AUC for M2BPGi level (AUC = 0.892) was superior to those for the FIB-4 (AUC = 0.818, *P* < 0.05), but the differences were not significant and were comparable to that or the APRI (AUC = 0.873, *P* = 0.598) and GPR (AUC = 0.851, *P* = 0.352). Overall, serum M2BPGi showed high reliability for the diagnosis of significant fibrosis and cirrhosis.

## Discussion

Early detection and intervention are critical for successes in disease curability. In chronic hepatitis C infection, it is quite critical to screen the patients for the status of liver fibrosis because those with advance fibrosis are at greater risk of developing severe complications [17–19]. Although a liver biopsy is the gold standard for staging of liver fibrosis, its invasive nature restricts its application in the clinic, especially in follow up cases. Therefore, the field of non-invasive approaches for liver staging has been recently evolved [20–23]. In the present study, we found that the serum M2BPGi levels as a simple and reliable glyco-biomarker diagnostic tool for staging of liver fibrosis in chronic hepatitis C patients (Fig. 2). The glycan-based immunoassay was previously developed as a simple system for automatically detecting unique fibrosis-related glyco-alterations [24–27]. To the best of our knowledge, the present study is the first study to evaluate the efficiency of serum M2BPGi in prediction of liver fibrosis stage in a large population of Chinese patients with HCV infection.

This study clearly demonstrated that the M2BPGi level in chronic hepatitis C patients increased with the progression of liver fibrosis stage (Fig. 2). This result came in a good agreement with previous studies [28–30]. Moreover, M2BPGi concentrations could also distinguish between healthy controls and hepatitis C patients, and this result suggests that alterations in serum M2BPGi may forecast liver disease. We also showed that M2BPGi level is an independent factor significantly associated with liver fibrosis in hepatitis C patients. Several other variables have been proposed to contribute to liver fibrosis, such as platelet count, total protein, ALP, GGT, total billirubin, HA, CIV, and AFP in our study (Table 2). Interestingly, among nine variables, including M2BPGi level, platelet count, total protein, ALP, GGT, total billirubin, HA, CIV, and AFP, M2BPGi level was the most significant serum marker associated with LSM.

Studies have showed that several risk scores such as the APRI, FIB-4, and GPR, which are calculated using the above variables, appeared to be a good surrogate marker for predicting fibrosis stages. Additionally, our results also showed that the M2BPGi level was strongly associated with other noninvasive fibrosis markers. As the AUCs of previously reported fibrosis markers (APRI, FIB-4, and GPR) increased by the progression of liver fibrosis stage, the AUC of M2BPGi increased accordingly as well. It worth to note that serum level of M2BPGi showed higher efficiency in predicting liver fibrosis grade F ≥ 2 than other surrogate markers, such as the FIB-4 and GDR (Table 3). Taken together, this suggests that the M2BPGi assay would be a good serological marker for predicting and differentiating liver fibrosis stags, especially the early and significant fibrosis stages.

On the other hand, M2BPGi level was also efficient in predicting cirrhosis (LSM > 15.3) with an AUC of 0.892 at a cut-off of 1.355, which is consistent with previous studies. In a study that included 106 Japanese patients with hepatitis C, cut-off values of M2BP for four fibrosis stages (F1–F4) ranged from 1.0 to 2.64 [31]. In another Japanese study performed on 137 primary biliary cirrhosis patients, performances were better than ours, with AUCs for significant fibrosis, severe fibrosis, and cirrhosis of 0.979, 0.933, and 0.965, respectively, and the cut off values for M2BPGi in fibrosis stages > F1, >F2, >F3, and > F4 were 0.7, 1.0, 1.4, and 2.0, respectively [14]. In our study, M2BPGi did not perform well for classifying patients at the extremes of significant and severe fibrosis (F2–F3). The reason may be coursed that Fibro Scan® is less accurate for detecting significant fibrosis (F2–F3) compared with Castera L [32].

The range of the M2BPGi serum thresholds observed in chronic hepatitis C patientswas different from those described in primary biliary cirrhosis (PBC) or non-alcoholic fatty liver disease [14, 33], typeIautoimmune hepatitis [34], and hepatitis B (Matsumoto and Umemura, personal communications). This difference might be contributed to the difference in patients’ ethnicity or due to the fact that the majority of our cohort was asymptomatic and exhibited mild fibrosis.

There are two main strengths of this study. First, relatively large sample size (*n* = 680) with well characterized baseline clinical characteristics. Second, in this study, we excluded patients who had fatty liver disease, which may have affected the Fibro Scan® value. On the other hand, the present study also has several limitations. First, we estimated the fibrosis stage using LSM and APRI or FIB4 in this study, while liver biopsy was not considered which could be a source of bias.

## Conclusions

Our study suggests that the level of serum M2BPGi would be a simple and reliable diagnostic tool for identifying liver fibrosis stage in Chinese patients with chronic hepatitis.

## Abbreviations

ALT: Alanine aminotransferase
anti-HBc: Antibody to hepatitis B core antigen
anti-HBsAg: Antibody to HBsAg
APRI: Aspartate transaminase to platelet ratio index
AST: aspartate aminotransferase
CI: Confidence interval
CIV: Type 4 collagen
ELISA: Enzyme-linked immunosorbent assay
FIB4: Fibrosis index based on four factors
GPR: Gamma-glutamyltranspeptidase to platelet ratio
HA: Hyaluronic acid
HBsAg: Hepatitis B surface antigen
HBV: Hepatitis B virus
HCV: Hepatitis C virus
IQR: Inter-quartile range
LN: Laminin
LSM: Liver stiffness measurement
M2BPGi: Mac-2 Binding Protein Glycosylation isomer
OR: Odds ratio
PBC: Primary biliary cirrhosis
RNA: Ribonucleic acid
VCTE: Vibration Controlled Transient Elastography™ system
WFA + -M2BP: Wisteria floribunda agglutinin-positive human Mac-2-binding AFP (α-fetoprotein)
